# Cognitive impairment and dementia—an update

**DOI:** 10.3389/fneur.2012.00153

**Published:** 2012-10-25

**Authors:** João Massano

**Affiliations:** ^1^Department of Neurology, Centro Hospitalar de São JoãoPorto, Portugal; ^2^Faculty of Medicine, Department of Clinical Neuroscience and Mental Health, University of PortoPorto, Portugal

Cognitive disorders have become a major theme in modern neuroscience. Analyzing the impact of these conditions at various levels, from personal to social and economic, it is not surprising that the amount of research on this subject has grown to vast figures in the past years, thus making it hard to keep up with the sum of publications and novelties in this field, especially for the busy clinician. On the other hand, basic and translational scientists frequently find it difficult to explore the vast and often complex clinical literature on this matter, as well as understand the clinical features of these disorders, in order to define the current research needs and optimal future directions in innovation. The main aim of the Frontiers Research Topic “Cognitive impairment and dementia—an update” is to deliver an updated synthesis regarding the current knowledge and literature in this field, which will hopefully benefit clinicians and scientists from various fields. In this regard, open access publication brings clear advantages. We have been very fortunate, as outstanding contributions from several authors and working groups have been submitted, covering a wide range of subjects.

Galimberti and Scarpini (2012) have approached the ever more complex subject of frontotemporal dementia genetics. This mini-review deals with the major issues regarding this topic, including genes involved, phenotypic aspects, and even the fresh scientific breakthrough in this field—the association between FTD and the pathologic hexanucleotide repeat expansion in the *C9ORF72* gene. This scientific novelty brought a long looked-for molecular explanation for a significant number of cases seen in clinic, especially those associated with amyotrophic lateral sclerosis and a positive family history.

Alves et al. (2012) have produced a comprehensive review concerning the most important aspects of Alzheimer's disease (AD), including clinical features, genetics, pathophysiology, clinical genetic testing, diagnostic strategies, and management. This article is very well complemented by the manuscript from de Mendonça (2012), who provides important reflections concerning the recent changing paradigms of clinical and scientific thinking in AD. Contributing also to the topic of AD, Sá et al. (2012) share their research on the neuropsychological aspects of early and late onset AD. This is a large series from one single group detaining extensive experience in this field.

Mild cognitive impairment and dementia have been recognized as important features of Parkinson's disease (PD) in the last few years, despite the traditional emphasis on motor symptoms of the disease (Massano, 2011; Massano and Bhatia, 2012). In this regard, Meireles and Massano (2012) have written a broad review concerning the issue of cognitive decline in PD, covering the stages of mild cognitive impairment and dementia. Phenotypic aspects have been approached, as well as diagnostic issues, genetic factors, and practical management strategies. The paper by Almeida (2012) delves further on the role of glucocerebrosidase in PD and other neurodegenerative conditions, a topic receiving currently much attention from basic and translational researchers, as well as clinicians. Other genes related to lysosomal functioning have also been dealt with in the text. The manuscript by Massano and Garrett (2012) complements the theme of cognitive impairment in PD by in-depth reviewing the literature regarding the cognitive effects of deep brain stimulation in these patients, and the importance of accurate preoperative cognitive assessment, as well as some ethical issues in this setting.

Neuropathology has traditionally been an important diagnostic instrument in cognitive disorders, and pathological findings have provided the basis for important genetic and pathophysiological lines of research along the years. However, this is a complex subject, especially in the context of neurodegenerative disorders, and non-pathologists find it difficult to keep up with the terminology. The article by Taipa et al. (2012) will be a precious aid to all of those interested in learning more about this subject or simply optimize their current level of knowledge, as it summarizes important neuropathological findings in the most common neurodegenerative dementias, establishing also a relationship with relevant clinical features.

Behavioral and psychological symptoms of dementia are a major source of disability and decrement in quality of life, as well as caregiver burden. Moreover, they are commonly difficult to tackle in practice, even for experienced clinicians. Cerejeira et al. (2012) have produced a very useful and comprehensive text on this subject, ranging from diagnosis to management, which will greatly benefit the readership.

Cognitive dysfunction in multiple sclerosis (MS) is a particularly sensitive issue, as this disease afflicts preferentially young adults, being one of the most important causes of neurologically induced disability in this age range. A few important ideas can be extracted from the paper by Guimarães and Sá (2012), such as the fact that, beyond every other symptoms of the disease, cognitive dysfunction in MS is a matter to keep in mind. Patients deserve proper assessment and management, as groundbreaking disease modifying therapies became available along the years.

Elderly people are naturally at higher risk of sustaining confusional states, especially those who suffer from previous brain disease leading to cognitive impairment. Unfortunately, delirium is still too often overlooked by clinicians, which brings onerous consequences to patients, since management opportunities are lost and outcome will be less favorable, as Martins and Fernandes point out in their manuscript (Martins and Fernandes, 2012). This is a very common disorder, which only stresses the importance of professional education and awareness on these matters. Broadly speaking, this is obviously another important aim of this Frontiers Research Topic.

Finally, an inclusive acknowledgment is due to the authors who, with their hard work, have contributed to this Frontiers Research Topic. In addition, reviewers should also here be appraised, as the manuscripts have clearly been improved after the successive comments posted on the review forums. Their honest efforts and purely altruistic commitment with this challenge have been truly admirable.
